# Survey dataset of building user-perceptions on the condition of public secondary school buildings in Ogun State Nigeria

**DOI:** 10.1016/j.dib.2018.06.108

**Published:** 2018-07-02

**Authors:** Oladunni Izobo-Matins, Abiodun Olotuah, Ekundayo Adeyemi, Kunle Ayo-Vaughan, Oluwole A. Odetunmibi

**Affiliations:** a Department of Architecture, College of Science and Technology, Covenant University, Nigeria; b Department of Mathematics, College of Science and Technology, Covenant University, Nigeria

**Keywords:** Building age, Building condition, Maintenance, User-perceptions

## Abstract

In this data article, questionnaire was administered to public secondary school teachers to investigate user-perceptions of the prevailing deterioration condition of the school buildings, in Ogun State, Nigeria. The condition of some factors such as, roof, paints, floor, walls, windows, doors, electrical, plumbing, toilets, WC, pipes, drains were the investigated variables. The data presented here are the opinion of the respondents. Through this research, it was discovered that most of the academic building of public secondary school of our study area were not properly managed and poorly maintained as a result of insufficient fund for maintenance and lack of maintenance culture.

**Specifications Table**

**Table:** 

Subject area	*Construction*
More specific subject area	*Building maintenance*
Type of data	*Tables, figure and Text files*
How data was acquired	*Through questionnaires and observation survey*
Data format	*Raw and analyzed*
Experimental factors	*Simple random sampling of existing public secondary school buildings in Ogun State, Nigeria*
Experimental features	*Computational analysis: Descriptive Analysis*
Data source location	*Ogun State, Nigeria*
Data accessibility	*Data is with this article*

**Value of the data**

• The data gives a basis for selection of walling materials according to the financial status of residents.
• It informs the government of the urgency in the maintenance needs of the public secondary school buildings.
• It revealed that majority of public secondary school buildings were old and few are middle aged buildings.
• The data may serve as a means of educating architects, who are designers of these school buildings, on the present condition of public school buildings.
• This data might offer insight on how to know and detect school buildings that are due for renovations.

## 1 Data

The data for this article were obtained from the survey research conducted in thirty- seven public secondary schools in Ogun state, Nigeria. It involves distribution of questionnaires to school principals and teachers who are users of the buildings. The questionnaire elicits responses on the present building conditions and expectations of the users in other to improve the teaching and learning performance of the students. The questionnaire also examined the length of stay of buildings in public secondary school, age of the public secondary schools involve in the research, most deteriorated academic building, deterioration factors from the user׳s perception, floor condition of the public school buildings and condition of water pipes in the public secondary schools.

### 1.1 Length of stay of buildings in public secondary school

**Fig. 1:**
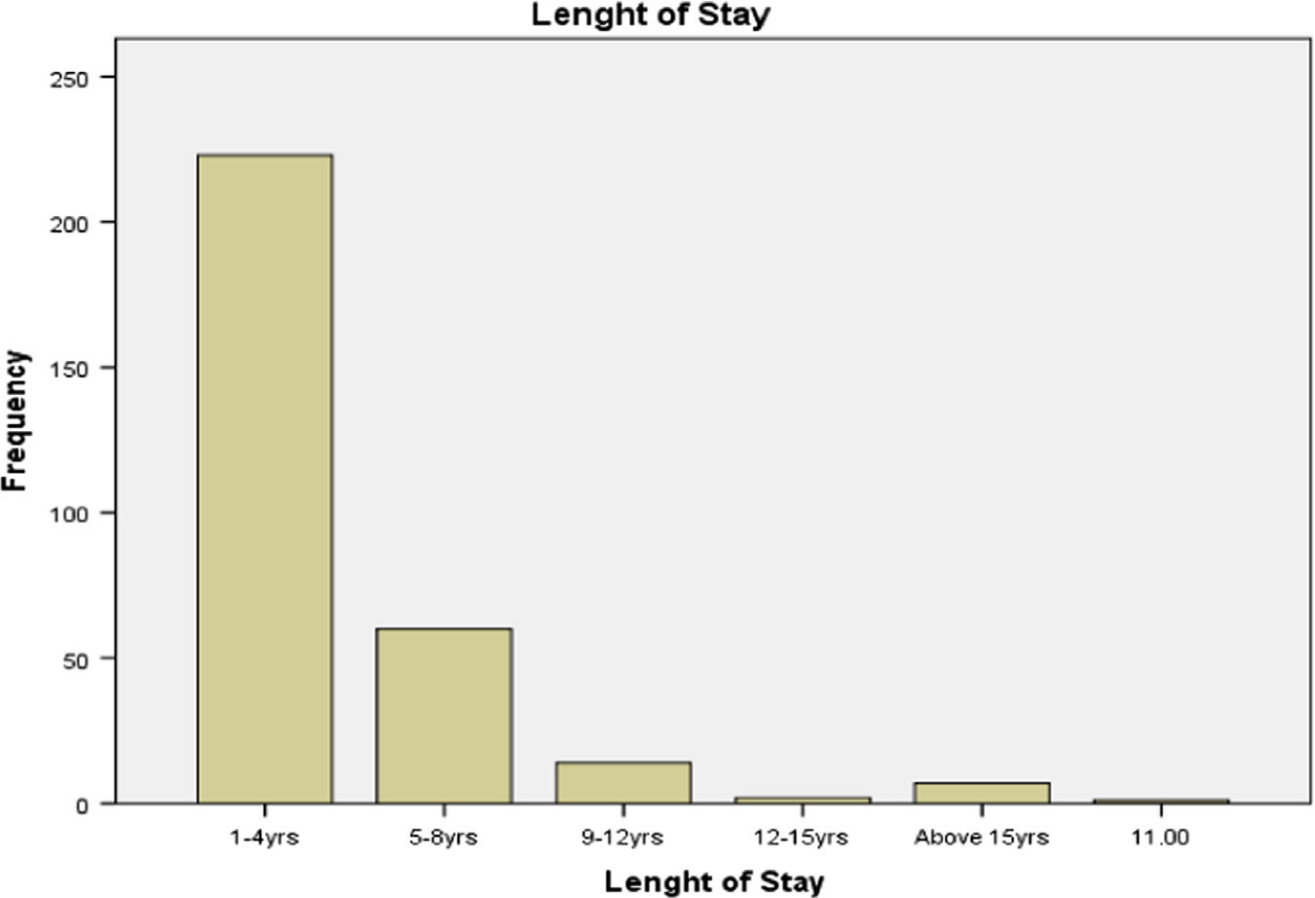
Bar chart of length of stay of buildings in public secondary schools in Ogun State.

Statistical summary of the length of stay of buildings in public secondary school available in our study area is presented in Table 1 below. It is discovered that length of stay of most of the buildings in public secondary schools in Ogun state examined for this research falls within 1 to 4 years. This suggests that, most of the buildings are newly built. The bar chart for the data is presented in Fig. 1.

**Table 1:** Length of stay of buildings in public secondary school.

Length of stay	Frequency	Valid percent
1–4 yrs	223	72.6
5–8 yrs	60	19.5
9–12 yrs	14	4.6
13–16 yrs	2	0.7
16 yrs and above	8	2.6
Total	307	100.0

### 1.2 Age of public secondary schools

Statistical summary of the age distribution (in terms of years they are established) of each of the schools involved in our research is presented in Table 2 below. It is discovered that most of the schools falls within 31 to 40 years and less than 20 years.

**Table 2:** Age of public secondary schools.

**School age**	**Frequency**	**Valid percent**
Up to 20	104	33.9
21–30	67	21.8
31–40	107	34.9
41–50	9	2.9
51 and above	20	6.5
Total	307	100.0

**Fig. 2:**
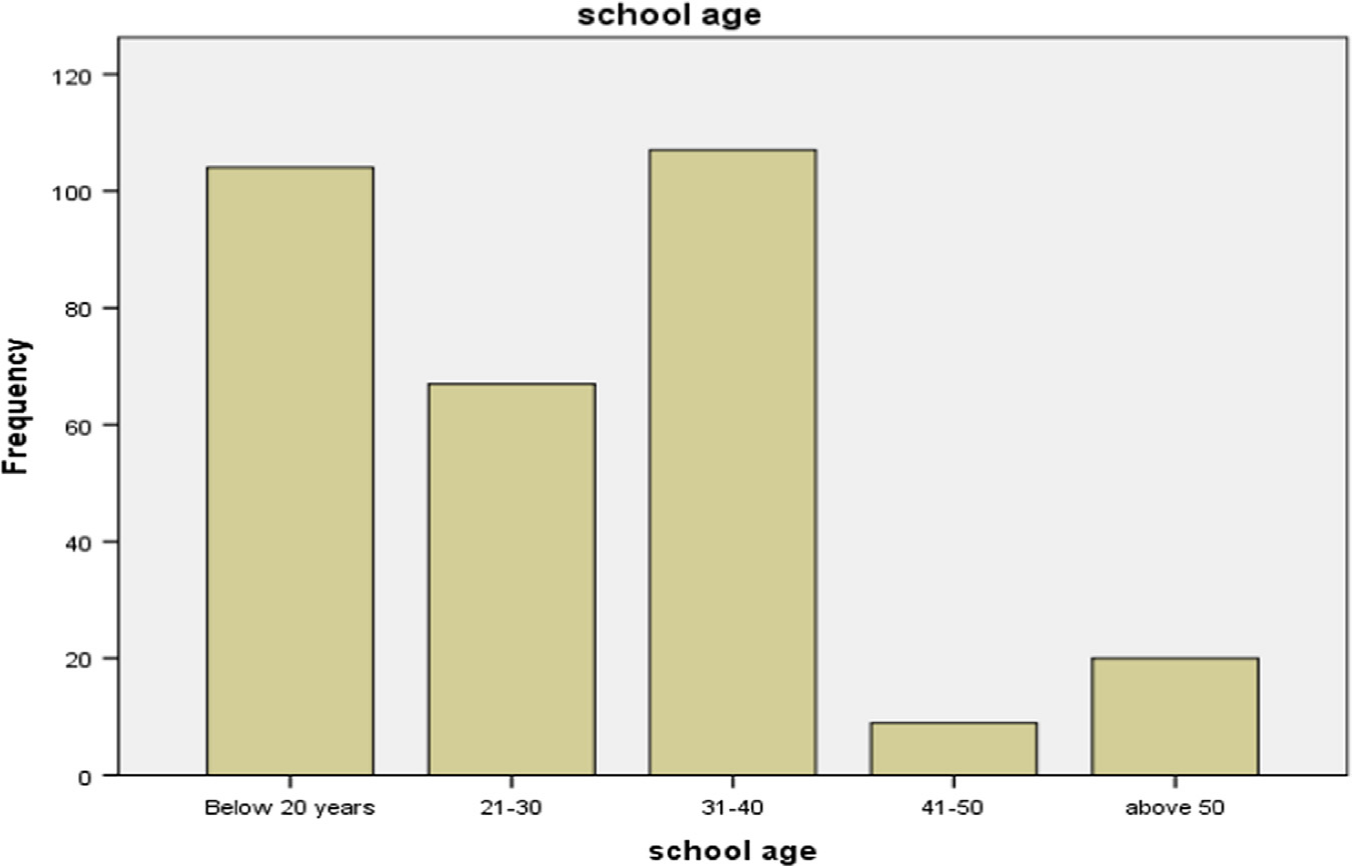
Bar chart of age of each of the schools.

Bar chart for the distribution is presented in Fig. 2.

### 1.3 Most deteriorated academic building

**Fig. 3:**
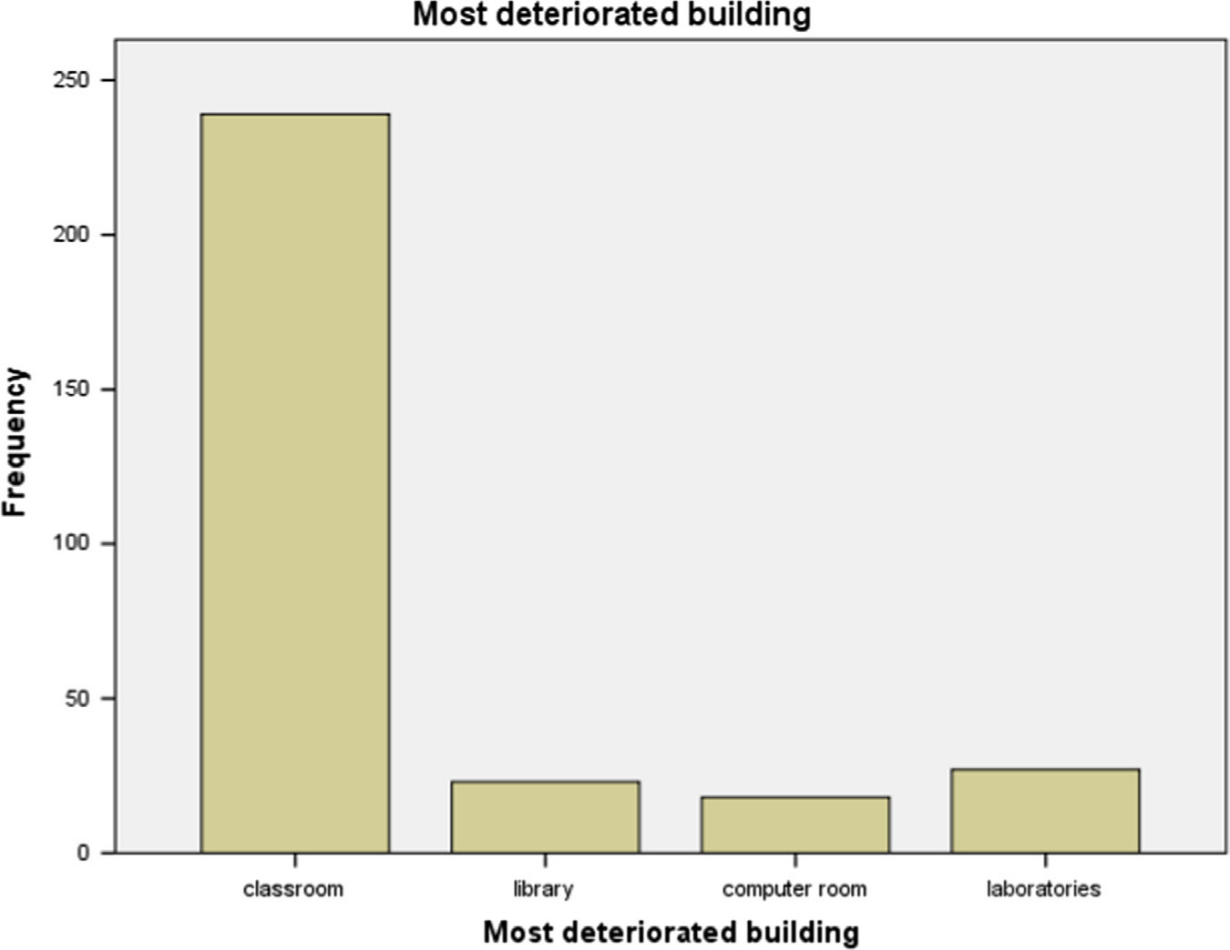
Bar chart for the most deteriorated building age of each of the schools.

Data on the most deteriorated academic building from our study area was collected and the statistical summary of the data is presented in Table 3 below. The result shows that buildings that are used for classroom are the most deteriorated. This could be as result of the frequent use of the building. The bar chart for the Table 3 is presented in Fig. 3.

**Table 3:** Mostly deteriorated academic building.

Most deteriorated academic building	Frequency	Valid percent
Classroom	239	77.9
Library	23	7.5
computer room	18	5.9
Laboratories	27	8.8
Total	307	100.0

### 1.4 Deteriorating factors from the users׳ perception

**Fig. 4:**
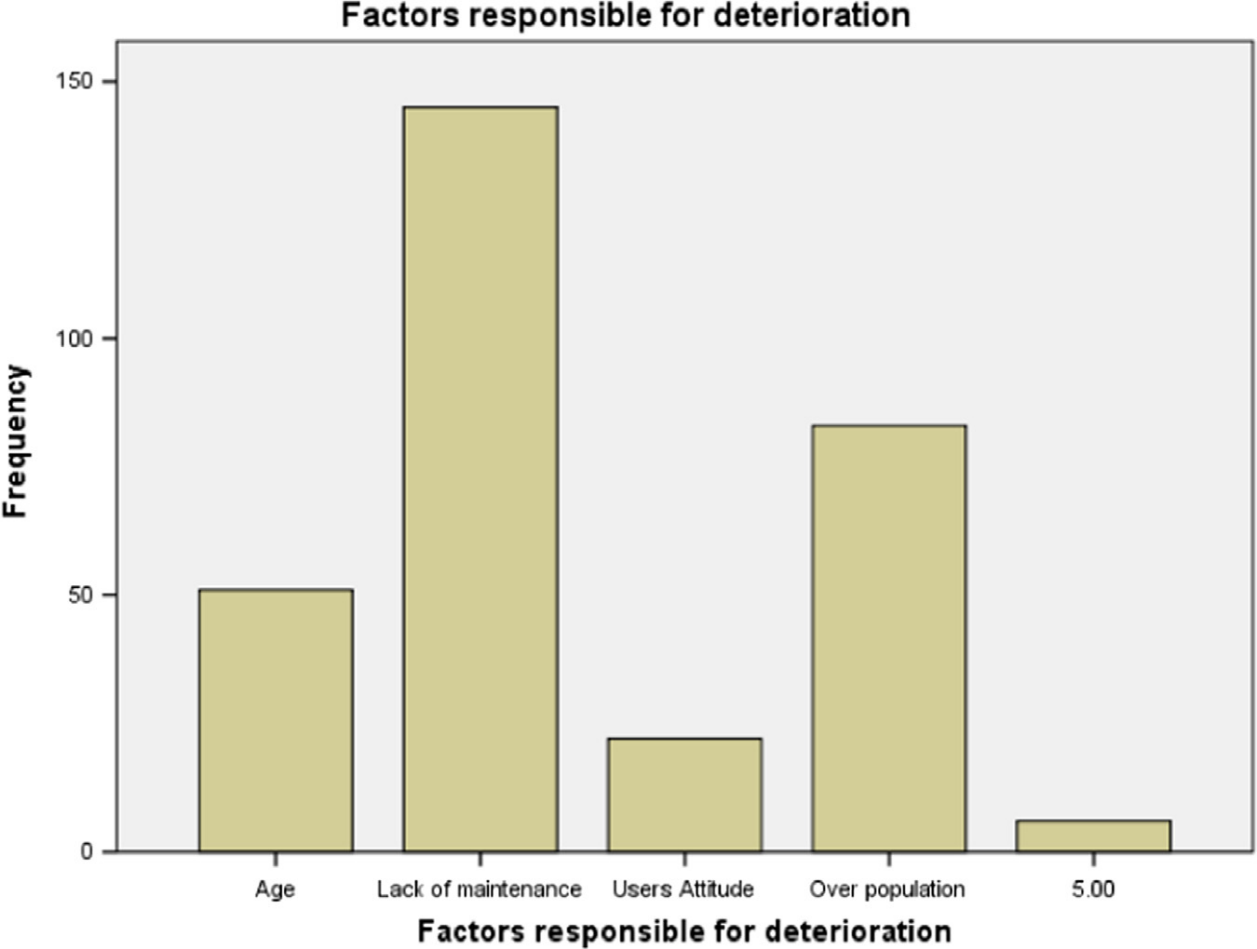
Bar chart for the factors that account for deterioration.

We examined factors that could be accounting for the deterioration of buildings from public schools in Ogun state from the users’ perspective. It was discovered that lack of maintenance as a result of insufficient fund for maintenance is the most significant factor. The result is presented in Table 4 below. The bar chart for the table is also presented in Fig. 4.

**Table 4:** Deterioration factors from users’ perception.

Deterioration factors	Frequency	Valid percent
Natural deterioration due to age	51	16.6
Lack of maintenance as a result Insufficient fund	145	47.2
Attitude of users and misuse of facilities	22	7.2
Over population and insufficient funding	89	29.0
Total	307	100.0

### 1.5 Walls condition of buildings in public secondary schools in Ogun State

**Fig. 5:**
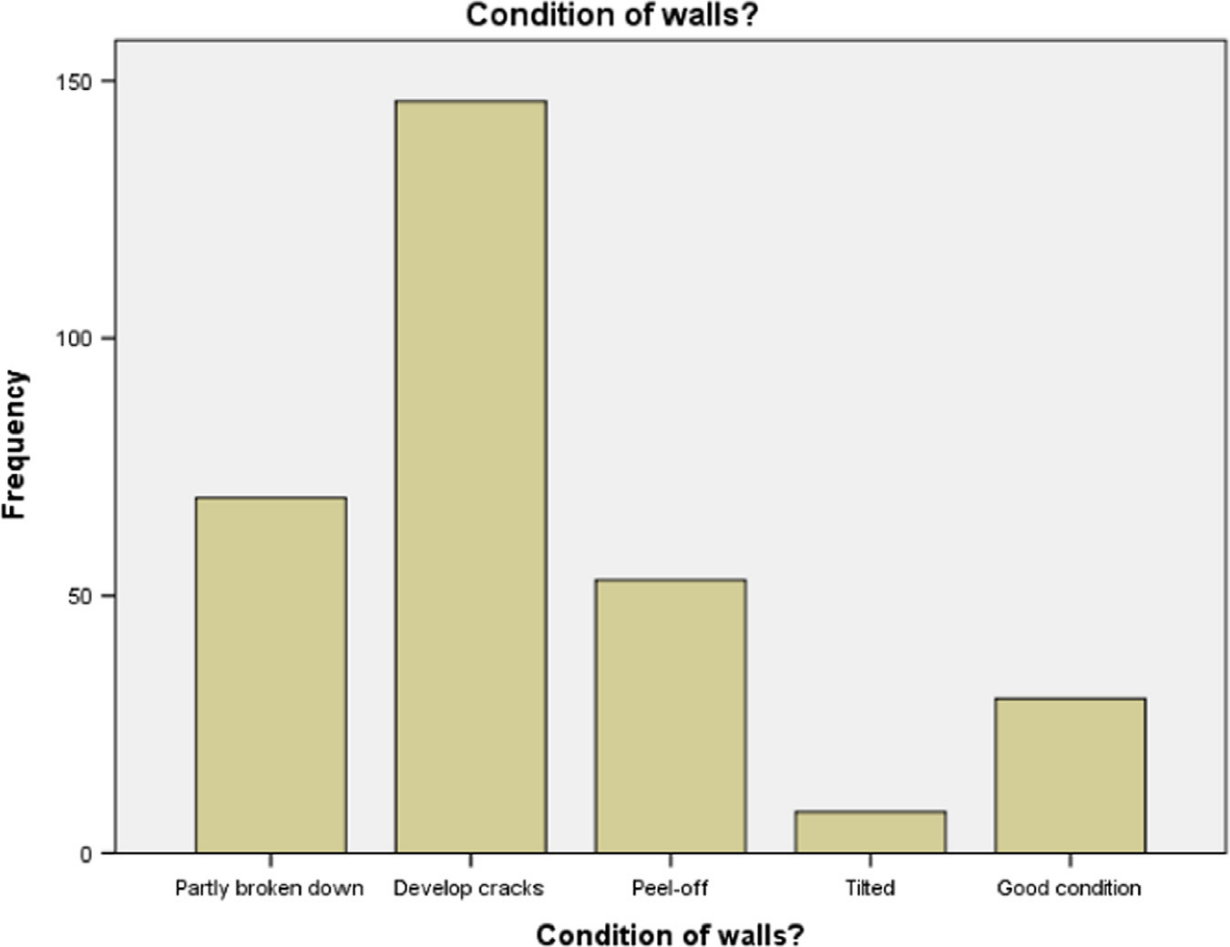
Bar chart for condition of walls.

Statistical summary of the condition of the walls of buildings from our study area is presented in Table 5. It is discovered that non-structural cracks is common among the schools used for our study. Bar chart for the distribution presented in Table 5 is presented in Fig. 5.

**Table 5:** Condition of walls.

Condition of Walls	Frequency	Valid percent
Structural (Tilted)	8	2.6
Partly broken down	69	22.4
Non-structural cracks	146	47.7
Good	84	27.3
Total	306	100.0

### 1.6 Condition of windows in the school buildings

**Fig. 6:**
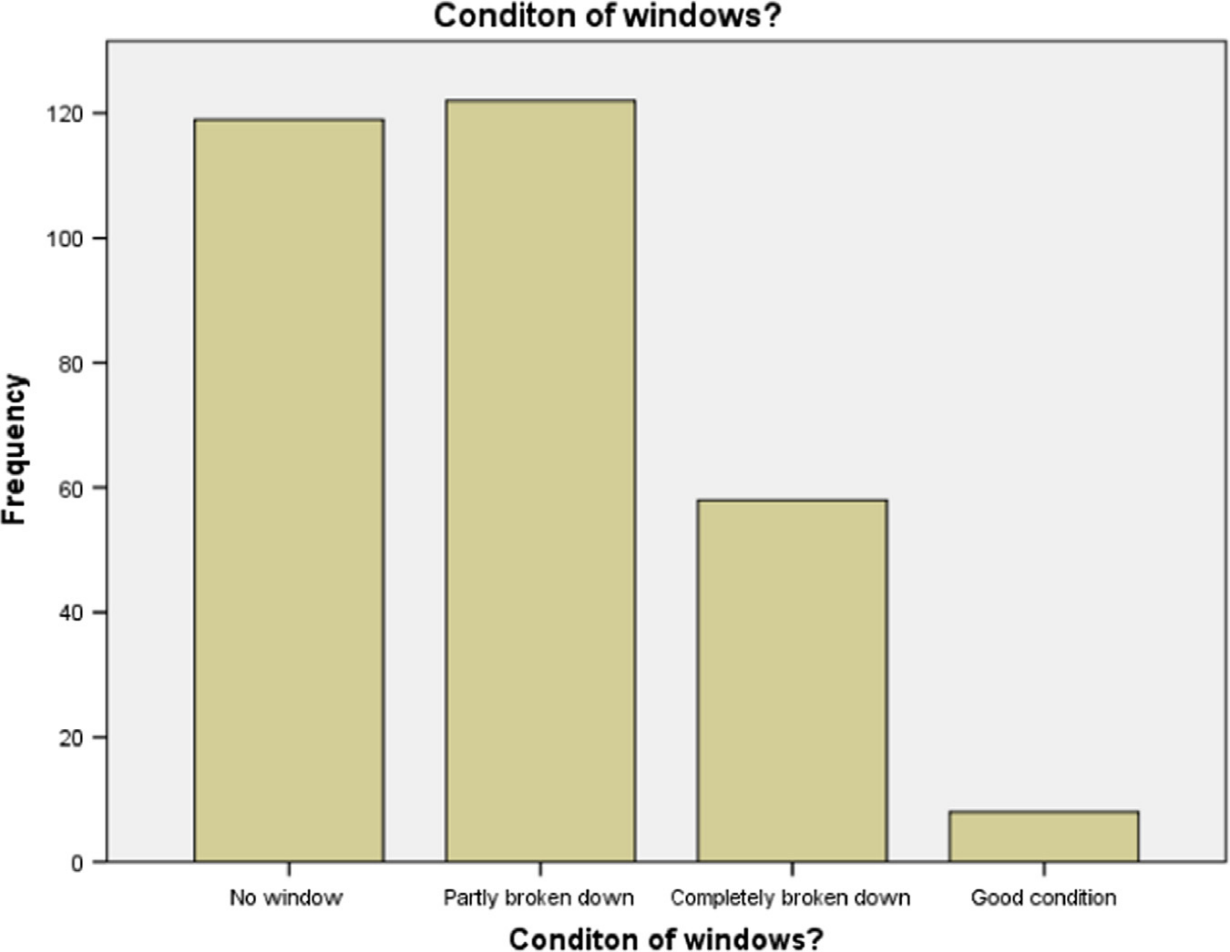
Bar chart for condition of windows.

Statistical summary of the condition of windows in the school used for our research is presented in Table 6 below. It is discovered that most of the windows has no louvre blades. Bar chart for the table presented in Fig. 6.

**Table 6:** Conditions of windows in the school buildings.

Window conditions of the buildings	Frequency	Valid percent
No Louvre blades just frame	119	38.8
Some glasses fall off	122	39.7
Completely broken down	58	18.9
In good shape	8	2.6
Total	307	100.0

### 1.7 Condition of doors in the academic buildings

**Fig. 7:**
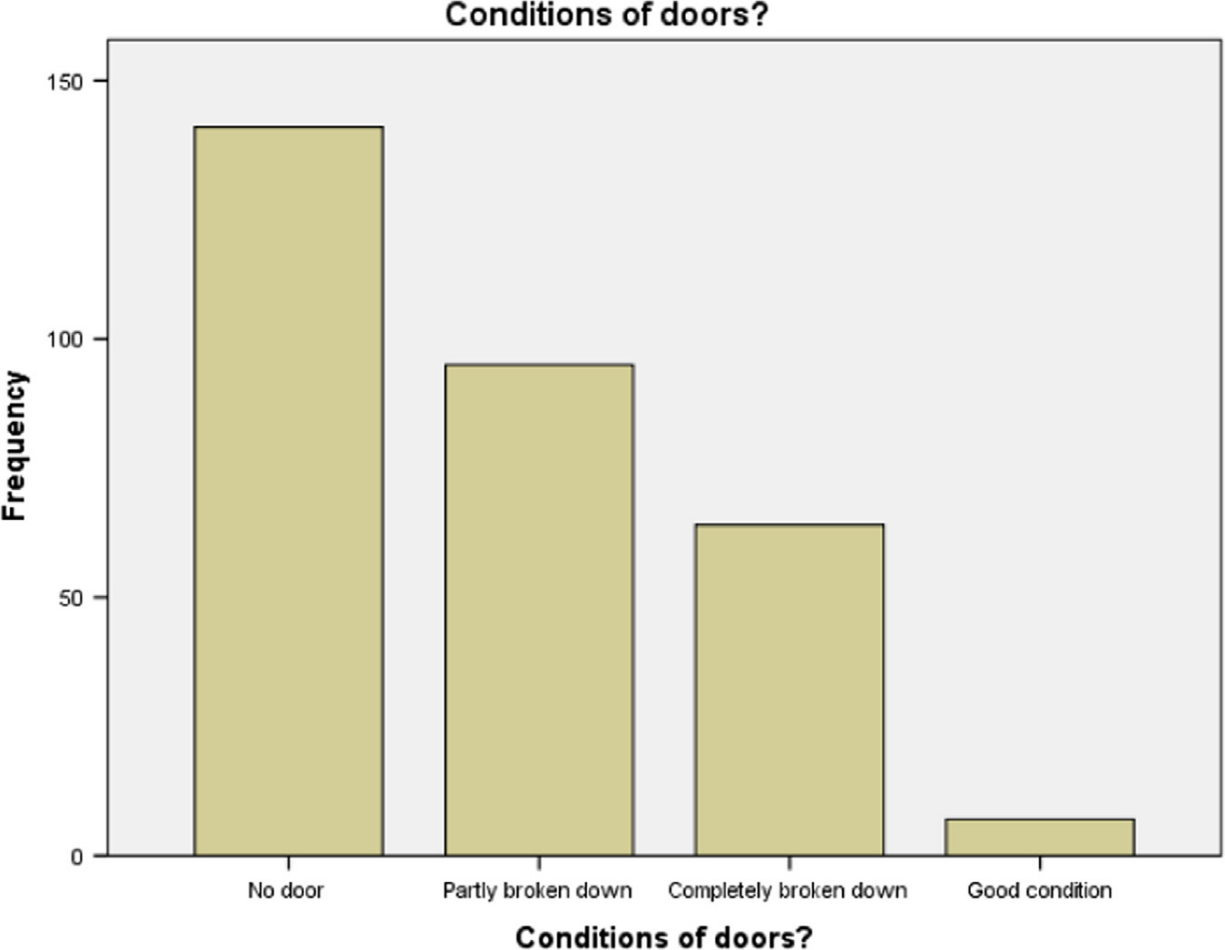
Bar chart for condition of doors.

Statistical summary for the condition of doors of all buildings that were used for our research is presented in Table 7 below. It is discovered that most of the buildings that were involved in this research work do not have doors again. Bar chart for the Table 7 is presented in Fig. 7.

**Table 7:** Conditions of doors in the academic buildings.

Condition of windows	Frequency	Valid percent
No door	141	45.9
Partly broken down	95	30.9
Completely broken down	64	20.8
Good	7	2.3
Total	307	100.0

### 1.8 Condition of water pipes in the public school in Ogun State

**Fig. 8:**
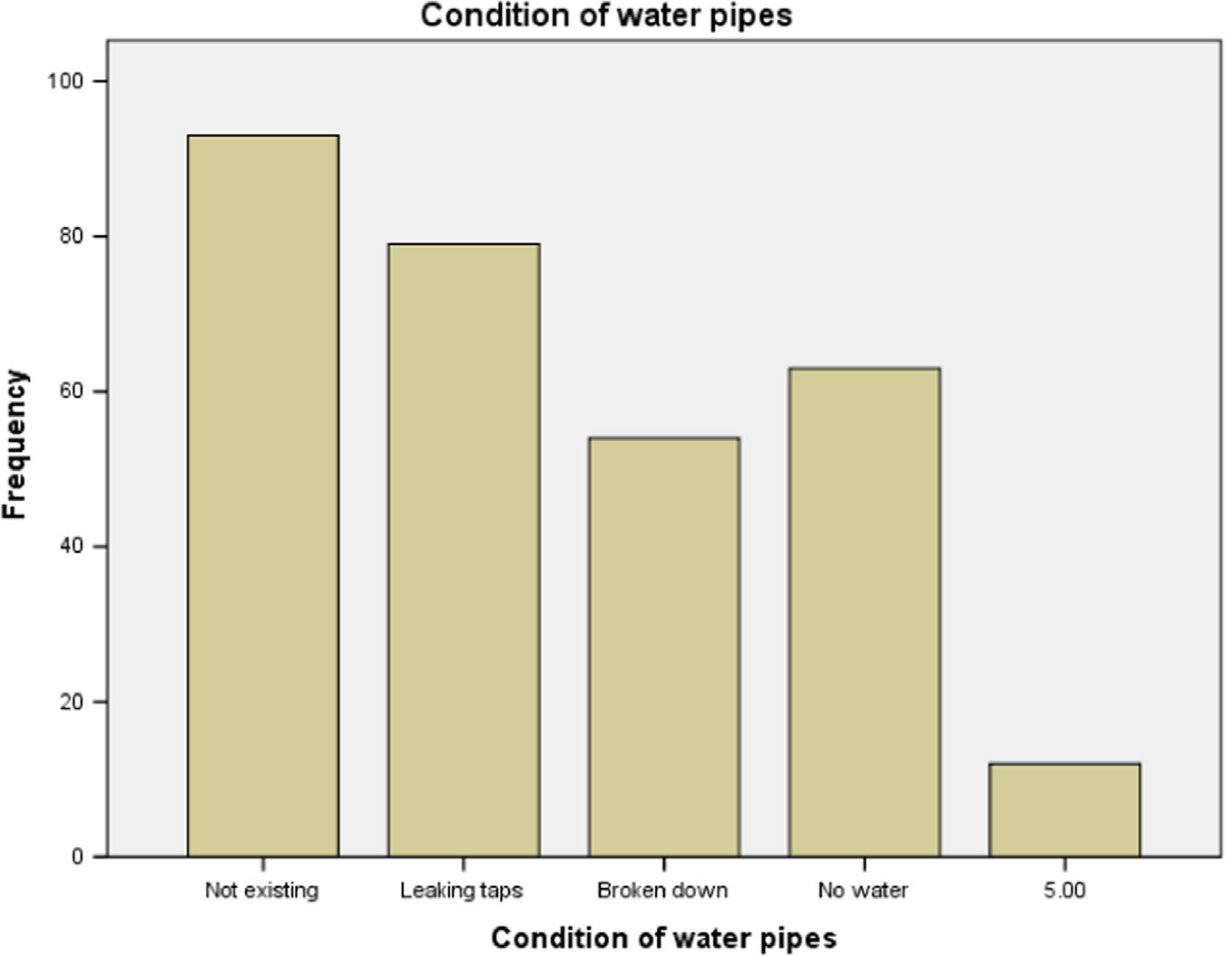
Bar chart for condition of water pipes.

We examined the condition of water pipes in the public secondary schools building that used for this research and the result is presented in Table 8 below. It is discovered that most of the water pipes of all the school buildings that were used for our research had broken down. Bar chart for the distribution is presented in Fig. 8.

**Table 8:** Condition of water pipes in the public secondary schools.

**Condition of water pipes**	**Frequency**	**Valid percent**
Minor defects in pipes	93	30.2
Leaking taps	79	25.7
Broken down	117	38.1
Good	18	6.0
Total	307	100.0

## 2 Experimental design, materials and methods

Several works have been done on condition and maintenance of public buildings [1], [2], [3], [4], [5], [6], [7], [8], [9], [10], [11], [12], [13], [14], [15], [16], [17], [18]. Careful selections of variables like roofs, paints, floor, walls, windows, doors, electrical, plumbing, toilets, WC, pipes, drains were investigated in this study. Three Hundred and seven (307) questionnaires were distributed by the researchers themselves and all the questionnaires were retrieved for further analysis. The questionnaires were purposively distributed to users who have spent nothing less than one years in a school because maintenance is periodical. The questionnaires were analysed using descriptive analysis methods.
