# Supplementary material for: Survey dataset of building user-perceptions on the condition of public secondary school buildings in Ogun State Nigeria
Source: Data Brief. 2018 Jul 2;19:2224–34. doi: 10.1016/j.dib.2018.06.108 (PMC6141440; doi:10.1016/j.dib.2018.06.108)

AUTHOR DECLARATION TEMPLATE

We wish to draw the attention of the Editor to the following facts which may be considered as

potential conflicts of interest and to significant financial contributions to this work. [OR]

We wish to confirm that there are no known conflicts of interest associated with this

publication and there has been no significant financial support for this work that could have

influenced its outcome.

We confirm that the manuscript has been read and approved by all named authors and that

there are no other persons who satisfied the criteria for authorship but are not listed. We

further confirm that the order of authors listed in the manuscript has been approved by all of

us.

We confirm that we have given due consideration to the protection of intellectual property

associated with this work and that there are no impediments to publication, including the

timing of publication, with respect to intellectual property. In so doing we confirm that we

have followed the regulations of our institutions concerning intellectual property.

We further confirm that any aspect of the work covered in this manuscript that has involved

either experimental animals or human patients has been conducted with the ethical approval

of all relevant bodies and that such approvals are acknowledged within the manuscript. [CAN

BE DELETED IF NOT RELEVANT]

We understand that the Corresponding Author is the sole contact for the Editorial process

(including Editorial Manager and direct communications with the office). He/she is

responsible for communicating with the other authors about progress, submissions of

revisions and final approval of proofs. We confirm that we have provided a current, correct

email address which is accessible by the Corresponding Author and which has been

configured to accept email from (oladunni.izobo-martins@covenantuniversity.edu.ng)

Signed by all authors as follows:

[1.] Dr. Izobo-Martins 17.05.18
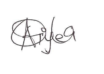


(2) Prof. Olotuah
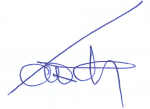

Supplement: Supplementary file 1 — Transparency document [file mmc1.docx]
